# Cognitive function mediates the relationship between age and anaesthesia-induced oscillatory-specific alpha power

**DOI:** 10.1093/braincomms/fcae023

**Published:** 2024-01-31

**Authors:** Gonzalo Boncompte, Isaac Freedman, Jason Qu, Isabella Turco, Zain Q Khawaja, Ignacio Cortinez, Juan C Pedemonte, Oluwaseun Akeju

**Affiliations:** Division of Anesthesiology, School of Medicine, Pontificia Universidad Católica de Chile, Santiago 8331150, Chile; Neurodynamics of Cognition Lab, School of Medicine, Pontificia Universidad Católica de Chile, Santiago 8331150, Chile; Department of Anesthesia, Critical Care and Pain Medicine, Massachusetts General Hospital, Harvard Medical School, Boston, MA 02114, USA; Department of Anesthesia, Critical Care and Pain Medicine, Massachusetts General Hospital, Harvard Medical School, Boston, MA 02114, USA; Department of Anesthesia, Critical Care and Pain Medicine, Massachusetts General Hospital, Harvard Medical School, Boston, MA 02114, USA; Department of Anesthesia, Critical Care and Pain Medicine, Massachusetts General Hospital, Harvard Medical School, Boston, MA 02114, USA; Department of Medicine, Case Western Reserve University School of Medicine, Cleveland, OH 44106, USA; Division of Anesthesiology, School of Medicine, Pontificia Universidad Católica de Chile, Santiago 8331150, Chile; Division of Anesthesiology, School of Medicine, Pontificia Universidad Católica de Chile, Santiago 8331150, Chile; Programa de Farmacología y Toxicología, Facultad de Medicina, Pontificia Universidad Católica de Chile, Santiago 8331150, Chile; Department of Anesthesia, Critical Care and Pain Medicine, Massachusetts General Hospital, Harvard Medical School, Boston, MA 02114, USA

**Keywords:** cognitive decline, aging, alpha oscillations, aperiodic activity, anaesthesia

## Abstract

Cognitive decline is common among older individuals, and although the underlying brain mechanisms are not entirely understood, researchers have suggested using EEG frontal alpha activity during general anaesthesia as a potential biomarker for cognitive decline. This is because frontal alpha activity associated with GABAergic general anaesthetics has been linked to cognitive function. However, oscillatory-specific alpha power has also been linked with chronological age. We hypothesize that cognitive function mediates the association between chronological age and (oscillatory-specific) alpha power. We analysed data from 380 participants (aged over 60) with baseline screening assessments and intraoperative EEG. We utilized the telephonic Montreal Cognitive Assessment to assess cognitive function. We computed total band power, oscillatory-specific alpha power, and aperiodics to measure anaesthesia-induced alpha activity. To test our mediation hypotheses, we employed structural equation modelling. Pairwise correlations between age, cognitive function and alpha activity were significant. Cognitive function mediated the association between age and classical alpha power [age → cognitive function → classical alpha; *β* = −0.0168 (95% confidence interval: −0.0313 to −0.00521); *P* = 0.0016] as well as the association between age and oscillatory-specific alpha power [age → cognitive function → oscillatory-specific alpha power; *β* = −0.00711 (95% confidence interval: −0.0154 to −0.000842); *P* = 0.028]. However, cognitive function did not mediate the association between age and aperiodic activity (1/f slope, *P* = 0.43; offset, *P* = 0.0996). This study is expected to provide valuable insights for anaesthesiologists, enabling them to make informed inferences about a patient’s age and cognitive function from an analysis of anaesthetic-induced EEG signals in the operating room. To ensure generalizability, further studies across different populations are needed.

## Teaser

Interpreting low intraoperative EEG alpha activity can be challenging due to its associations with both age and cognitive function. However, Boncompte *et al*. have skilfully addressed this issue by showing that cognitive function plays a significant role in mediating the relationship between age and the oscillatory component of alpha activity.

## Graphical Abstract

**Graphical Abstract:**
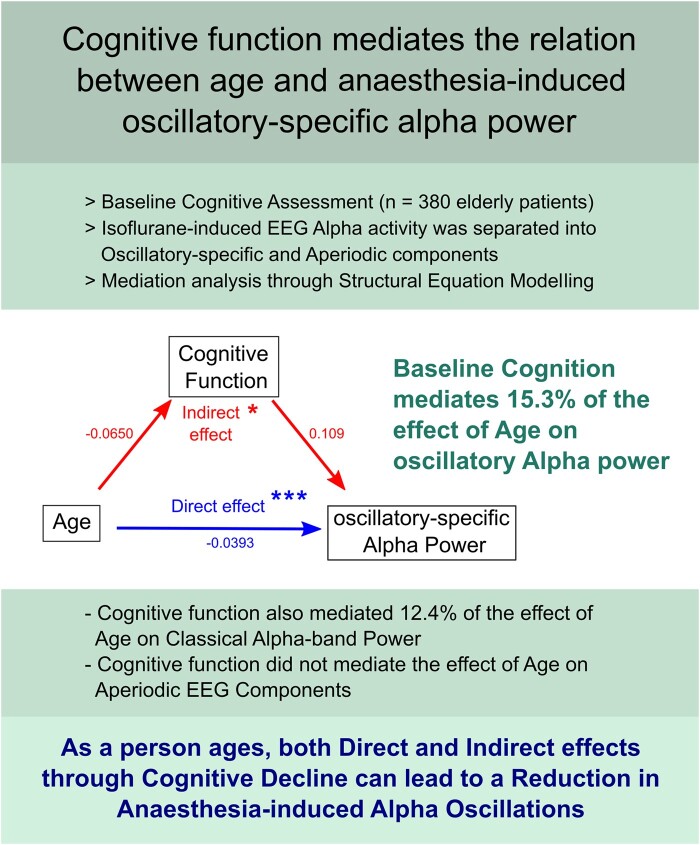

## Introduction

Anaesthetic drugs are crucial to modern medicine, allowing for safe and comfortable surgical procedures. Although the mechanisms by which these drugs induce the state of general anaesthesia are not fully understood, research has shown that anaesthetic drugs induce structured EEG activity that varies based on the drug class and dose used.^1-3^ Studies of general anaesthesia maintained with inhaled anaesthetic vapours (desflurane, isoflurane and sevoflurane) have shown that these medications induce slow (0.1–4 Hz), frontal theta (4–8 Hz) and frontal alpha (8–12 Hz) band activities.^3^ Biophysical and animal models have suggested that the frontal alpha oscillations are a thalamocortical rhythm caused by increased gamma aminobutyric acid-A (GABA-A) receptor conductance and decay time.^4-7^ Clinically, frontal alpha oscillations are widely accepted as a neurophysiological marker of an anaesthetized state in which recall of intraoperative events is not likely.

However, the relationship between anaesthetic drugs and EEG oscillations is complex. Recent research has shown that several patient-specific factors, such as aging, cognition and comorbidities, influence alpha power.^8-11^ This complexity, and others (i.e. aging is independently associated with a decline in some cognitive domains^12^), highlights the need for further research into how patient-specific factors interact with the generation of alpha activity. The relationship between alpha and cognition is of particular interest; preoperative cognitive decline has been linked to poor surgical outcomes such as increased likelihood of complications and postoperative delirium.^13^ However, our understanding of the relationship between intraoperative alpha power, aging and cognition is incomplete, partly because the collinearity between aging and cognition has been largely unaccounted for in studies.

Alpha power, or the magnitude of alpha oscillations, is classically quantified as the (mean or peak) spectral power within the alpha range (∼8–12 Hz).^14^ However, recent literature has emphasized that this measure [classical alpha (clAlpha)] is the sum of alpha oscillations [oscillatory-specific alpha (osAlpha)] and background aperiodic activity.^15-18^ Aperiodic activity refers to patterns of activity that do not occur in a specific frequency (e.g. 10 Hz) but throughout a wide range of frequencies (e.g. 0.1–40 Hz). EEG signals showcase aperiodic activity that follows a power-law or scale-free behaviour.^16^ Importantly, osAlpha and aperiodic activity are not equivalent and are related to distinct clinical and neurobiological processes.^19^ Here, we tested the hypothesis that cognitive function mediates the relation between aging and osAlpha power. With this, we aim to improve our understanding of the sources of variability of brain activity observed during anaesthesia and thus help anaesthesiologists better interpret EEG activity patterns in the operating room.

## Materials and methods

This research study was approved by the Partners Human Research Committee (Institutional Review Board 20168000742) and is a sub-study of the Minimizing ICU Neurological Dysfunction with Dexmedetomidine-induced Sleep (MINDDS) trial.^20^ The MINDDS trial was a double-blinded, placebo-controlled, single-site, parallel-arm study investigating the use of a postoperative sleep-inducing dose of dexmedetomidine for preventing delirium in elderly patients undergoing major cardiac surgery. This sub-study analysed data from participants who underwent preoperative assessments and had intraoperative EEG recordings. The data reported here were obtained either preoperatively (cognitive function) or intraoperatively (EEG) and thus were not influenced by the postoperative administration of dexmedetomidine. This work is reported in accordance with the AGReMA guidelines for reporting mediation studies.^21^

### Study population

**Figure 1:**
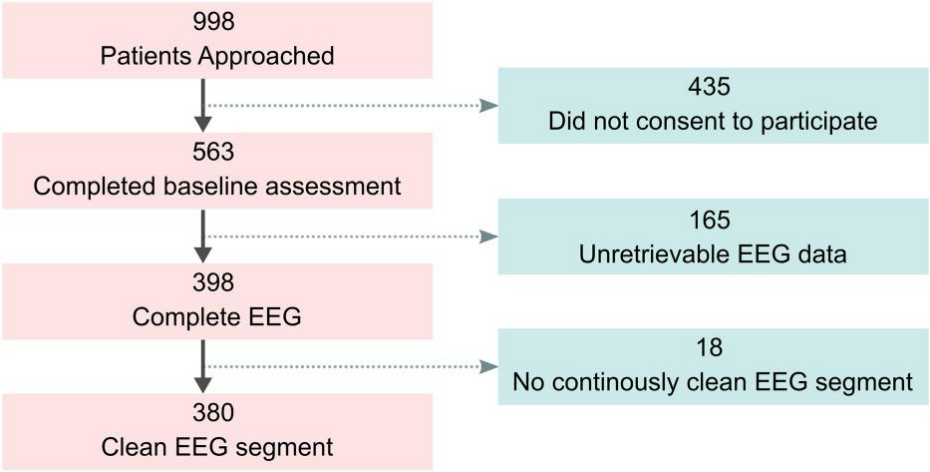
**Consort diagram.** It illustrates the steps taken for cohort selection and the number of patients.

The details of the MINDDS study, including inclusion and exclusion criteria, have previously been published.^20^ Briefly, patients were eligible for inclusion if they were 60 years or older, scheduled to undergo a cardiac surgical procedure with cardiopulmonary bypass and were scheduled for a same-day surgical admission. Patients were excluded from participation if they had renal (requiring dialysis) or liver failure (Child–Pugh score > 5), were on chronic benzodiazepine or antipsychotic therapy, had severe deficit(s) due to structural or anoxic brain damage or were SARS-CoV-2 positive or symptomatic (e.g. fever, cough and loss of taste/smell). Participants who were blind, deaf or unable to communicate in English were excluded due to their inability to complete the cognitive assessments, as were patients experiencing circumstances for which long-term follow-up might be difficult. All participants gave written informed consent. Data from 380 patients were analysed in this study (see Fig. 1 for further details).

### Data collection

Patients were recruited between March 2017 and July 2021. Baseline cognitive function was assessed during a pre-randomization assessment using the telephonic Montreal Cognitive Assessment (t-MoCA), a validated screening tool adapted from the MoCA that has demonstrated adequate sensitivity in screening for mild cognitive impairment (MCI). This validation has been done by equivalence testing with other tools that assess MCI.^22^ t-MoCA scores range from 0 (worst) to 22 (best) points. Although t-MOCA is not a diagnostic tool, a value equal to or below 17 points suggests the presence of MCI.^22^

We recorded EEG data using the SedLine monitor (Masimo Inc., Irvine, CA). SedTrace electrode arrays were placed on the forehead at approximately Fp1, Fp2, F7 and F8, the ground electrode at approximately Fpz and the reference electrode ∼1 cm above Fpz. Data were recorded with a sampling frequency of 250 Hz. General anaesthesia was induced with an intravenous induction agent, followed by maintenance with isoflurane according to clinical criteria. We selected EEG data segments using information from the electronic medical record and spectral analysis. For each patient, we visually inspected the raw EEG signal and its spectrogram to select a 2-min artefact-free segment during the maintenance phase of general anaesthesia, at least 15 min after induction and before the onset of cardiopulmonary bypass.

### Spectral estimations

Clean EEG segments of each patient were used to estimate the median spectral power within the alpha frequency band (8–12 Hz, clAlpha power), osAlpha activity (osAlpha power) and aperiodic activity. To estimate clAlpha power in each electrode, each 2-min segment was divided into overlapping (95%) 3-s windows. The power spectral density (PSD) of each window was calculated using multitappers (seven DPSS tappers, Python MNE library^23^). Afterwards, a single PSD per subject was obtained as the median value of each frequency across windows. Then, clAlpha power for that electrode was calculated as the median value within the alpha frequency range. We then averaged the clAlpha power across electrodes to obtain a single value per patient.

To obtain osAlpha power and the aperiodic components, we employed the specparam algorithm (also known as FOOOF^18^). This algorithm models each PSD as a sum of an aperiodic ‘1/f’ component and oscillatory components. First, it finds the best estimates for aperiodic activity using a power-law equation (1):


(1) $$Power=Offset*Frequency^{-Slope}$$

where offset and slope are free parameters. Then, it attempts to fit oscillatory activity as a Gaussian on top of the aperiodic activity. The algorithm iteratively repeats these two steps until the best model is found. PSD estimates for specparam were obtained similarly to the PSD employed for clAlpha estimation except for a larger window (4 s) and with 99% overlap to improve smoothness. These PSDs were used in the specparam algorithm to obtain aperiodic slope, offset and oscillatory peak power within the alpha frequency range. Specparam parameters used were as follows: peak_width_limits = 0.5 to 5; max_n_peaks = 3; peak_threshold = 2; aperiodic_mode = “fixed”. Peaks (oscillations) were allowed in the models if they occurred from 0.5 to 40 Hz but only considered alpha oscillations if their centre frequency was between 8 and 12 Hz.

### Statistical analysis

Table 1 provides a summary of the participant characteristics. To analyse discrete variables, we used *χ*^2^ or Fisher’s exact test, while for continuous variables, the Mann–Whitney U-test was employed. Spearman correlations were used for pairwise correlations, and the *Z*-test of the Fisher-transformed rho values, following a previously established strategy,^24^ was used to compare correlation coefficients. Mediation analyses were performed using structural equation models implemented in the ‘mediation’ R package,^25^ with 5000 bootstrapped iterations to estimate 95% confidence intervals (CIs). Statistical significance was determined using a threshold value of *α* = 0.05. Mediation effect size was assessed using the mediation proportion.^26^ All statistical analyses, except mediation analyses, were conducted using JASP software.

**Table 1:** Patient’s characteristics separated by cognitive function

	MCI (t-MoCA ≤ 17)	No MCI (t-MoCA > 17)	*P*-value
*N*	105	274	
Demographics			
Age (median [IQR])	71.00 [66.00, 77.00]	68.50 [64.00, 73.00]	**0**.**001**
Sex (%)			
F	22 (21.0)	78 (28.5)	0.175
M	83 (79.0)	196 (71.5)	
Height (cm; median [IQR])	175.30 [167.6, 180.3]	175.30 [167.60, 180.30]	0.967
Weight (kg; median [IQR])	85.95 [72.75, 100.85]	85.50 [74.15, 95.75]	0.5
Body mass index (median [IQR])	28.29 [24.62, 32.00]	27.77 [25.03, 30.83]	0.365
Education (%)			**0**.**0028**
High school or less	19 (23.2)	27 (12.2)	
Some college/associate degree	21 (25.6)	45 (20.3)	
Bachelor’s degree	22 (26.8)	67 (30.2)	
Master’s or doctorate degree	20 (24.4)	83 (37.4)	
Previous diagnostics			
Hypertension (%)	72 (87.8)	167 (74.9)	**0**.**023**
Peripheral artery disease (%)	6 (7.3)	17 (7.6)	1
Cerebrovascular disease (%)	10 (12.2)	25 (11.2)	0.971
Liver disease (%)	4 (4.9)	5 (2.2)	0.41
Patient-reported outcomes measures (PROMIS)			
Physical function (median [IQR])	45.50 [39.12, 50.40]	46.40 [40.10, 52.50]	0.487
Global physical (median [IQR])	50.80 [42.30, 57.70]	50.80 [44.90, 57.70]	0.66
Cognitive (median [IQR])	49.50 [43.52, 61.23]	53.00 [46.80, 62.70]	0.06
Global mental (median [IQR])	56.00 [48.30, 62.50]	56.00 [50.80, 62.50]	0.791
Pain (median [IQR])	40.70 [40.70, 55.37]	40.70 [40.70, 53.20]	0.135
Sleep (median [IQR])	48.40 [43.80, 54.30]	50.50 [43.80, 54.30]	0.49
Surgery characteristics			
Surgery duration (median [IQR])	6.02 [5.24, 6.73]	5.70 [5.09, 6.47]	0.077
Type of procedure (%)			0.099
AV replacement	19 (23.2)	45 (20.2)	
AV replacement + CAB	13 (15.9)	21 (9.4)	
AV replacement + MV	2 (2.4)	4 (1.8)	
CAB only	18 (22.0)	42 (18.8)	
MV repair	10 (12.2)	48 (21.5)	
MV repair + CAB	2 (2.4)	8 (3.6)	
MV replacement + CAB	3 (3.7)	1 (0.4)	
MV replacement only	0 (0.0)	7 (3.1)	
Other	15 (18.3)	47 (21.1)	
EEG features			
clAlpha power (median [IQR])	2.77 [1.51, 4.61]	3.76 [2.25, 6.21]	**0**.**001**
osAlpha power (median [IQR])	7.12 [5.73, 8.78]	7.75 [6.44, 9.65]	**0**.**018**
Offset (median [IQR])	2.04 [1.66, 2.37]	2.16 [1.86, 2.46]	**0**.**049**

IQR, interquartile range; AV, aortic valve; CAB, coronary artery bypass; MV, mitral valve. Bold numbers mark *p* values lower than 0.05.

## Results

Here, we analysed data from 380 elderly patients, only one of which lacked basal t-MoCA assessment. Table 1 presents an overview of participant characteristics stratified by t-MoCA scores. Patients that screened positive for MCI had, on average older, lower educational levels and a higher incidence of hypertension. No other clinical variable showed differences between groups.

### Pairwise correlations

**Figure 2:**
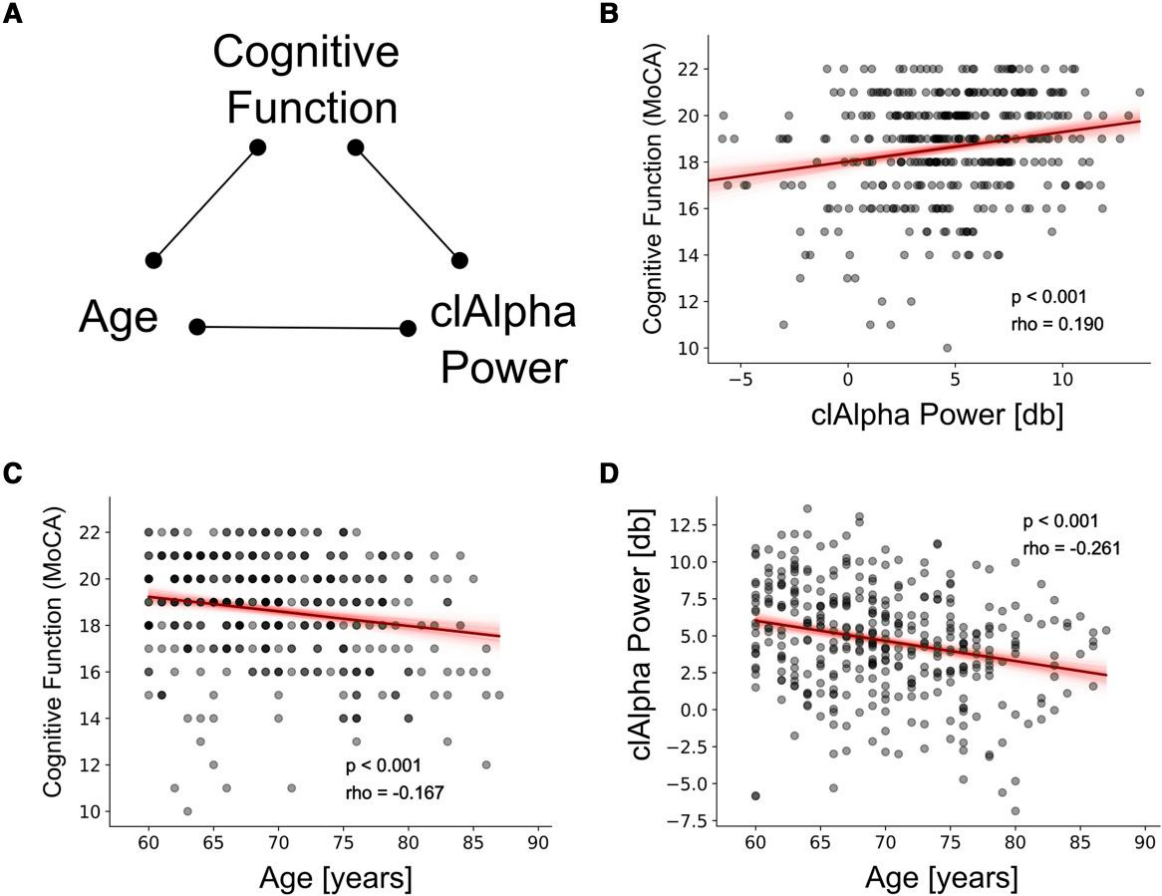
**Pairwise correlations between age, cognitive function and clAlpha power.** (**A**) Illustration showing all paired combinations between the variables of interest. (**B**) Scatter plot depicting the significant pairwise correlation between clAlpha and cognitive function. (**C**) Scatter plot depicting the significant pairwise correlation between age and cognitive function measured by MoCA (telephone version of the MoCA test). (**D**) Scatter plot depicting the significant pairwise correlation between age and clAlpha power. Thick lines show the best linear regression, and the shade depicts the 95% CI of the regression for illustrative purposes. Statistical assessments of the correlations were done using Spearman correlation.

Figure 2A demonstrates all possible paired correlations between three variables of interest. A positive correlation was observed between cognitive function and clAlpha power [rho = 0.190 (95% CI: 0.091–0.285), *P* < 0.001, Fig. 2B]. This suggests that patients with higher baseline cognitive function exhibited greater clAlpha power during anaesthesia. On the other hand, increasing age was associated with lower preoperative cognitive function [rho = −0.167 (95% CI: −0.264 to −0.068), *P* = 0.0011, Fig. 2C]. Similarly, increasing age was associated with lower clAlpha power [rho = −0.261 (95% CI: −0.352 to −0.165), *P* < 0.001, Fig. 2D].

**Figure 3:**
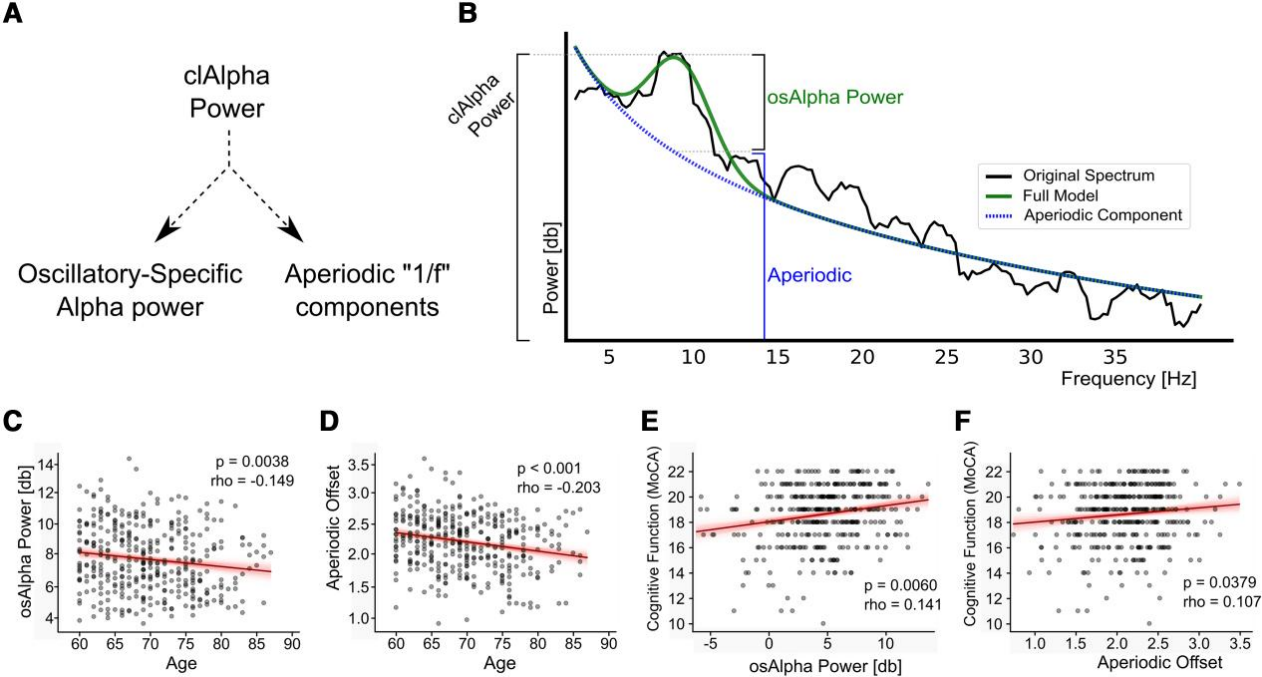
**Pairwise correlations between age, cognitive function and the two components of clAlpha activity, osAlpha power and aperiodic activity.** (**A**) Illustration showing that clAlpha power can be decomposed into osAlpha power and an aperiodic component. (**B**) Graphical depiction of how the ‘specparam’ algorithm carries out this separation. It shows a representative PSD with a fitted aperiodic power-law decay and an oscillatory-specific component modelled as a Gaussian function. (**C–F**) Scatterplots showing the significant correlations between (**C**) age and osAlpha power, (**D**) age and offset, (**E**) osAlpha power and cognitive function measured using the telephone version of the MoCA and (**F**) offset and cognitive function. Thick lines show the best linear regression, and the shade indicates the 95% CI for the regression for illustrative purposes. Statistical assessments of the correlations were done using Spearman correlation.

Subsequently, we conducted separate analyses for the constituent components of clAlpha power (Fig. 3A and B). Significant correlations were observed between age and osAlpha power [rho = −0.149 (95% CI: −0.246 to −0.049), *P* = 0.0038, Fig. 3C] and aperiodic offset [rho = −0.203 (95% CI: −0.297 to −0.104), *P* < 0.001, Fig. 3D]. Similarly, significant correlations were observed between cognitive function and osAlpha power [rho = 0.141 (95% CI: 0.041–0.239), *P* = 0.0061, Fig. 3E] and aperiodic offset [rho = 0.107 (95% CI: 0.006–0.201), *P* = 0.038, Fig. 3F].

### Mediation analyses

**Figure 4:**
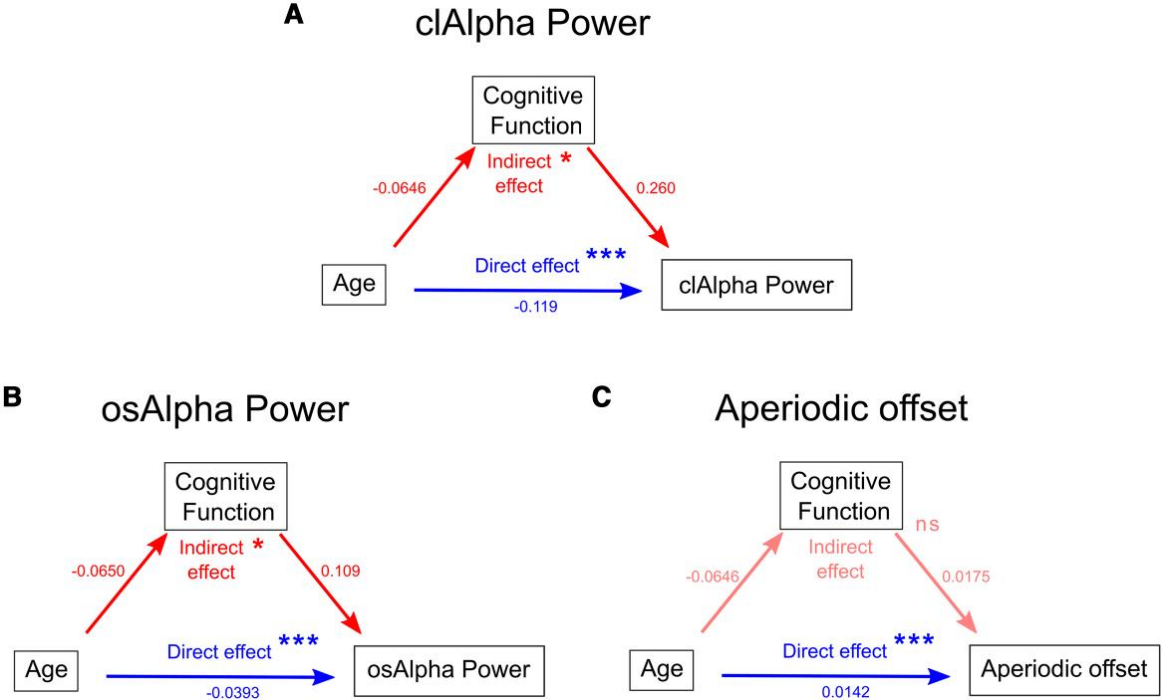
**Path diagrams of mediation analyses. A** shows the path diagrams of a mediation model in which clAlpha power is explained by direct (only by age) and indirect (age influences cognitive function, which in turn affects clAlpha power) paths. **B** and **C** correspond to analogous models in which age and cognitive function explain osAlpha power and aperiodic offset instead of clAlpha power. Numbers beside each arrow correspond to the regression coefficients (beta values). Red symbolizes indirect effects, and blue symbolizes the direct effects of the mediation analyses. *P < 0.05 and ***P < 0.001. ns, non-significant effects.

We conducted a mediation analysis using structural equation modelling, with age as the predictor, clAlpha power as the outcome and preoperative cognitive function as the mediator of the association between age and clAlpha power (Fig. 4A). We found both a significant direct effect [age → clAlpha; *β* = −0.119 (95% CI: −0.177 to −0.0648); *P* < 0.001] and indirect effect [age → cognitive function → clAlpha; *β* = −0.0168 (95% CI: −0.0313 to −0.00521); *P* = 0.0016], indicating that cognitive function mediated the effect that age has on clAlpha power. This mediation comprised 12.4% (95% CI: 3.5–27.2%) of the total effect of age on clAlpha. When osAlpha power was used instead of clAlpha power in a mediation model (Fig. 4B), the direct effect [age → osAlpha power; *β* = −0.0393 (95% CI: −0.0727 to −0.00626); *P* = 0.0196] and the indirect effect [age → cognitive function → osAlpha power; *β* = −0.00711 (95% CI: −0.0154 to −0.000842); *P* = 0.028] were significant. The mediation proportion of cognitive function towards osAlpha power was 15.3% (95% CI: 1.33–57.5%). In a third model that employed aperiodic offset as the predicted variable (Fig. 4C), the direct effect [age → offset; *β* = −0.0142 (95% CI: −0.0216 to −0.0071), *P* < 0.001] was significant, while the indirect mediation effect [age → cognitive function → offset; *β* = −0.00113 (95% CI: −0.00287 to 0.00019); *P* = 0.0996] was not significant. We also conducted a mediation model employing aperiodic slope, a related component of aperiodic activity. Similarly, in the aperiodic slope model, the direct effect [age → slope; *β* = −0.0779 (95% CI: −0.0123 to −0.0032); *P* < 0.001] was significant, while the indirect effect [age → cognitive function → slope, *β* = −0.00035 (−0.0014 to 0.00045), *P* = 0.43] was not significant.

## Discussion

In this work, we analysed EEG data from a large cohort of patients that underwent isoflurane-induced general anaesthesia. Our findings replicated previous research, supporting associations between age, preoperative cognitive function and intraoperative alpha activity.^8,10,12,27,28^ Using structural equation modelling in this triple relation, we show that cognitive function acts as a mediator of the relation between age and alpha activity. Additionally, we investigated two components of classically measured alpha band power, namely oscillatory-specific activity and aperiodic activity. We found that cognitive function acts as a mediator between age and oscillatory-specific activity but not for the effect of age on aperiodic EEG activity.

The number of years since birth is insufficient to explain the neurobiological changes that underpin age-related cognitive decline because aging is highly heterogeneous; some people retain full cognitive capacities past their 90s, and others show a significant cognitive decline in their 60s.^12,29^ The particular factors and mechanisms determining the speed of age-related cognitive decline still need to be fully understood. However, cardiovascular, inflammatory and other neurobiological factors are thought to play crucial roles.^30^ Two critical neurobiological processes linked to age-related cognitive decline are a marked reduction of cerebral volume that occurs predominantly in the frontal lobe^29,31^ and white matter deterioration that is observable in MRI as hyperintensities.^32^ These processes are consistent with a reduction in the capacity of the thalamocortical circuits to produce GABAergic anaesthesia-induced alpha oscillations.^5,7^

Although classically confounded, periodic and aperiodic components of EEG spectral activity relate to different cognitive processes in health and pathology (also see Merkin *et al.*^33^). Recent evidence has started to describe the clinical utility of the aperiodic component of EEG. For example, recent work indicates that aperiodic activity can distinguish children with attention deficit and hyperactivity disorder from neurotypical controls.^34^ More related to our work, aperiodic 1/f slope has been shown to predict schizophrenia more accurately than neural oscillations or behavioural results.^35^ Importantly, both components have a distinct neurobiological basis. Oscillatory activity is the result of temporally coordinated postsynaptic potentials arising from spiking activity that is precise and rhythmical.^36^ Aperiodic activity, in contrast, is thought to reflect the broad balance between glutamatergic and GABAergic neuronal activity, the balance between excitatory and inhibitory cortical activity.^17,37,38^ This idea has been supported by the fact that inhibitory anaesthetic agents like propofol and isoflurane systematically modify the 1/f slope (increasing low-frequency while concomitantly decreasing high-frequency activity).^39,40^ It is important to mention that a relation between aperiodic parameters and age has been previously described.^41-43^ In this context, our results emphasize the importance of better understanding cortical activity under different regimes, such as GABAergic anaesthetics, which could provide insights into the integrity of cortical circuits and their dynamical responsiveness.

A limitation of the present study is that cognitive function was assessed using the t-MoCA test. It is important to remark that cognitive function refers to a wide range of mental abilities, and not all behave in the same way across aged individuals. It has been proposed that more ‘fluid reasoning’ declines earlier and faster than ‘crystalized knowledge’ type mental skills.^12^ Another limitation of the present study was that it was a sub-study, and the parent trial had inclusion (e.g. age >60) and exclusion (e.g. renal failure requiring dialysis). Thus, our results may not be entirely generalizable, and future studies are needed. Our findings highlight the joint impact of age and cognitive function on isoflurane-induced alpha power. However, it is essential to note that these factors do not explain all the variance in alpha power. Therefore, while age and cognitive function are crucial to interpreting low intraoperative alpha activity, other unidentified factors and unmeasured confounders (e.g. inflammation and anaesthetic dose) may play a significant role. Similarly, this was an observational study, and thus, we do not claim our results demonstrate causal relations between age, cognitive function and alpha activity. Further investigations are warranted to gain a comprehensive understanding of the mechanisms underlying intraoperative alpha oscillations.

This research analysed EEG data from patients undergoing isoflurane-induced general anaesthesia and identified significant connections between age, preoperative cognitive function and intraoperative alpha activity. The findings indicated that aging is associated with decreased intraoperative alpha activity and cognitive decline, with cognitive function mediating the association between age and osAlpha activity. This study is expected to provide valuable insights for anaesthesiologists, enabling them to make informed inferences about a patient’s age and cognitive function from an analysis of anaesthetic-induced EEG waveforms in the operating room.

## Data Availability

Data are available upon reasonable request.
